# Use of *RNALater*^®^ Preservation for Virome Sequencing in Outbreak Settings

**DOI:** 10.3389/fmicb.2017.01888

**Published:** 2017-09-26

**Authors:** Claudia Kohl, Merle Wegener, Andreas Nitsche, Andreas Kurth

**Affiliations:** Centre for Biological Threats and Special Pathogens, Robert Koch Institute, Berlin, Germany

**Keywords:** metagenome, virome, outbreak, *RNAlater*, Paramyxovirus

## Abstract

Outbreaks of infectious diseases may occur in animal and human populations; this underlines the need for suitable One Health approaches. During outbreak situations, straightforward identification of etiological agents is indispensable for taking countermeasures. A recently published protocol for metagenomic virus detection in clinical specimens (TUViD-VM) was developed for snap-frozen tissues which can be challenging to obtain. Here, we describe the use of *RNALater^®^*-treated tissue at ambient temperatures for virome sequencing. This study demonstrates that samples stored in *RNALater^®^* buffer yield similar results to those stored snap-frozen.

## The Study

Identification of etiological agents is of high importance when assessing outbreak situations. One of the challenges in One Health diagnostic is that detection methods are often only validated and available for human samples or samples from certain animal species. When these specific virus detection approaches fail, unbiased sequencing methods are often used to increase the chance of pathogen detection. So far, approaches for virus enrichment prior to sequencing are being based on native snap-frozen samples (Kohl et al., 2015). This is due to the fact that most of the protocols are using any kind of mechanical separation step between the actual virus particles and undesired background. Mechanical separation or enrichment (i.e., ultracentrifugation, filtration, centrifugation or digestion of background genome) requires intact virus particles. Particle structures are best preserved at very low temperatures. In the field, these cooling requirements are sometimes challenging as liquid nitrogen or dry ice is often difficult to obtain, hard to transport or not available at all. However, even if snap-freezing were available in the field, the transportation of the samples at such low temperatures via aircraft would remain a further costly challenge.

To overcome these difficulties, we evaluated the application of the TUViD-VM protocol on samples preserved in *RNALater^®^* RNA Stabilization Reagent (Qiagen, Hilden, Germany). *RNALater^®^* is widely used for tissue archiving and stabilization of RNAs. Qiagen states that tissue RNA is protected in *RNALater^®^* for 7 days at 15–25°C, for 4 weeks at 2–8°C and for longer periods at -20°C or -80°C (^1^last accessed 17th of August 2017). Additionally, it has been shown that the protection of RNA in *RNALater^®^* at ambient temperatures is equivalent to that by snap-freezing in liquid nitrogen (Mutter et al., 2004; Wang et al., 2006). Moreover, other studies showed that *RNALater^®^* may also be able to preserve the infectivity of viral particles (and therefore the structure) at ambient conditions over a longer period of time (Uhlenhaut and Kracht, 2005; Kurth, 2007). In this study we evaluated the use of *RNALater^®^*-preserved tissues stored at ambient temperatures as starting material for unbiased virus purification protocols and metagenomic sequencing (TUViD-VM).

Embryonated chicken eggs were infected with Sendai virus (SeV) strain Hei, and livers were extracted, homogenized and validated as described before (Kohl et al., 2015).

**FIGURE 1:**
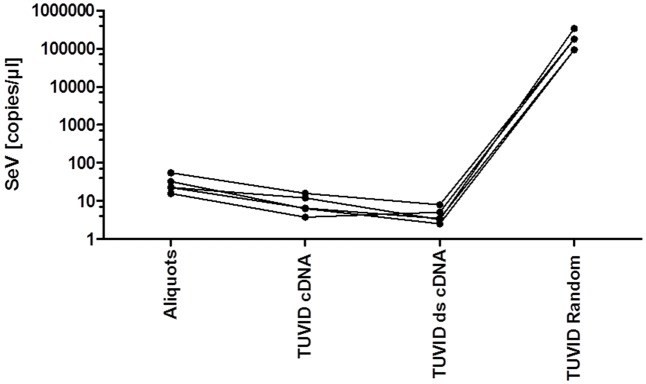
Reproducibility and variation during the TUViD-VM purification method. Duplicates were measured for the presence of SeV RNA during different stages of TUViD-VM purification to evaluate the reproducibility of the method.

**FIGURE 2:**
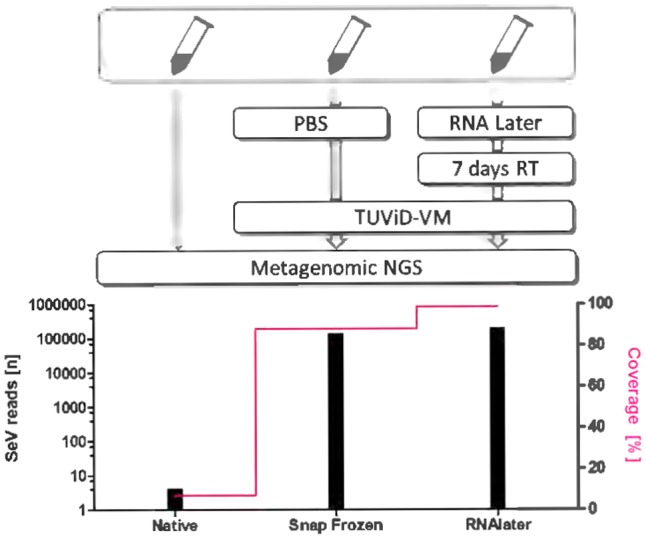
Description of the purification procedure for comparative NGS. All preparations started with the SeV infectious liver tissue aliquots prepared and validated before. A native aliquot was forwarded to NGS without additional purification procedure. A snap-frozen aliquot was thawed and diluted with 100 μl of PBS and purified by TUViD-VM before NGS as described before. An *RNALater^®^* aliquot was thawed and diluted with 100 μl of *RNALater^®^* and kept at ambient conditions for 7 days before TUViD-VM and NGS. The left *Y* axis indicates the total number of SeV reads obtained by NGS, normalized to a total of 1,000,000 reads. The right *Y* axis indicates the genome coverage of SeV, revealed by mapping the reads to the reference genome of SeV (accession number KT120023). This likely underestimates the true number of reads present by possibly unmapped reads due to rapid mutation rate of RNA viruses and thus divergence from the selected reference genome. RT, Room temperature.

Liver homogenates (52 copies/μl for SeV) were stored in aliquots of 100 μl at -80°C. First, the integrity of the aliquots was evaluated. Additionally, the reproducibility of the TUViD-VM method was demonstrated in four independent preparations shown in **Figure 1**. For validation of the TUViD-VM in *RNALater^®^* samples, these aliquots were used either snap frozen or stored in *RNALater^®^* at ambient temperature and prepared in duplicate as depicted in **Figure 2**.

The samples were analyzed for the presence of SeV and the TATA-box-binding protein (TBP) housekeeping gene prior to sequencing as described previously (Kohl et al., 2015). For the native/non-enriched sample duplicates, the CT values were similar (SeV 52 copies/μl, TBP 226 copies/μl), the snap-frozen duplicates and the *RNALater* duplicates showed similar results (SeV 1.3^∗^10^7^ copies/μl, TBP = 0 and 1.0^∗^10^7^ copies/μl, TBP = 0), respectively. For metagenomic sequencing, one of the equal duplicates was randomly picked and sequenced on the Personal Genome Machine (PGM) and analyzed as described for the TUViD-VM protocol (Kohl et al., 2015). The total number of SeV reads in the snap-frozen sample was 136,441 and in the *RNALater* sample 198,715, respectively. These reads covered 88.1 percent and 98.8 percent of the SeV genome, respectively (**Figure 2**). In comparison, the native sample, not undergoing further purification, resulted in 4 SeV reads (6 percent of SeV genome).

We demonstrated the feasibility of using *RNALater^®^*-stored tissue homogenates (and possibly other fluid sample material) as basis for metagenomic sequencing. PCR and sequencing results for the *RNALater^®^*-stored samples are similar to results obtained with snap-frozen samples. Investigators are aiming to preserve their samples in the best way possible. Using snap-frozen samples in field settings and outbreak investigations is the predominant procedure. Snap-freezing is often challenging as it requires extra equipment and costly transportation. Dry ice and liquid nitrogen has to be available to keep samples at ultra-low temperatures. Dry shippers are a safe and lasting way to transport samples in liquid nitrogen, but beside the problem of unavailability of liquid nitrogen, the shipper has to be charged in advance. Also it can be difficult and expensive to transport the shipper on a passenger plane, due to dangerous goods regulations and heavy weight. Often only transport via air freight is possible, at even higher costs. We showed that *RNAlater^®^* storage can help to overcome these drawbacks for metagenomic sequencing. *RNALater^®^*-preserved tissue homogenates can be stored for at least 7 days at ambient temperature without any loss in detectability. However, a study conducted by Riesgo et al. (2012) on preservation of sponge tissue described a potential storage limit of even 1 month for RNA preserved samples. Storing samples in *RNAlater^®^* at ambient temperatures will also increase the possible amount of samples for transportation during outbreak situations, which would be of further benefit in the context of One Health. This all together represents a major advantage for researchers using metagenomic sequencing in virus discovery, outbreak settings and One Health.

## Biographical Sketch

CK is a scientist at the Robert Koch Institute in Berlin Germany at the Centre for Biological Threats and Special Pathogens. Her research interests are the detection of emerging and re-emerging viruses and the characterization of novel pathogens.

## Author Contributions

Designed experiments: CK, AN, and AK. Conducted experiments: CK and MW. Analyzed the data: CK. Wrote the publication: CK, MW, AN, and AK.

## Conflict of Interest Statement

The authors declare that the research was conducted in the absence of any commercial or financial relationships that could be construed as a potential conflict of interest.

## Footnotes

1 https://www.qiagen.com/de/resources/resourcedetail?id=19988eb0-6df5-4d26-8435-33efe98197da&lang=en
